# Case management for frail older people – a qualitative study of receivers’ and providers’ experiences of a complex intervention

**DOI:** 10.1186/1472-6963-14-14

**Published:** 2014-01-10

**Authors:** Magnus Sandberg, Ulf Jakobsson, Patrik Midlöv, Jimmie Kristensson

**Affiliations:** 1 Department of Health Sciences, Faculty of Medicine, Lund University, P.O. Box 157, Lund SE-221 00, Sweden; 2 The Swedish Institute for Health Sciences (Vårdalinstitutet), Lund University, P.O. Box 187, Lund SE-221 00, Sweden; 3 Center for Primary Health Care Research, Faculty of Medicine, Lund University, Malmö SE-205 02, Sweden; 4 Department of Clinical Sciences in Malmö, Faculty of Medicine, Lund University, Malmö SE-205 02, Sweden

**Keywords:** Case management, Frail elderly, Intervention studies, Qualitative evaluation

## Abstract

**Background:**

Case management interventions have been widely used in the care of frail older people. Such interventions often contain components that may act both independently of each other and interdependently, which makes them complex and challenging to evaluate. Qualitative research is needed for complex interventions to explore barriers and facilitators, and to understand the intervention’s components. The objective of this study was to explore frail older people's and case managers’ experiences of a complex case management intervention.

**Methods:**

The study had a qualitative explorative design and interviews with participants (age 75-95 years), who had received the case management intervention and six case managers who had performed the intervention were conducted. The data were subjected to content analysis.

**Results:**

The analysis gave two content areas: providing/receiving case management as a model and working as, or interacting with, a case manager as a professional. The results constituted four categories: (1 and 2) case management as entering a new professional role and the case manager as a coaching guard, as seen from the provider’s perspective; and (3 and 4) case management as a possible additional resource and the case manager as a helping hand, as seen from the receiver’s perspective.

**Conclusions:**

The new professional role could be experienced as both challenging and as a barrier. Continuous professional support is seemingly needed for implementation. Mutual confidence and the participants experiencing trust, continuity and security were important elements and an important prerequisite for the case manager to perform the intervention. It was obvious that some older persons had unfulfilled needs that the ordinary health system was unable to meet. The case manager was seemingly able to fulfil some of these needs and was experienced as a valuable complement to the existing health system.

## Background

Complex interventions are challenging to evaluate since they contain components that may act both independently of each other and interdependently, which makes it complicated to assess individual aspects of the intervention. The challenges are, among others, related to difficulties in standardising the study design and delivery of the intervention, and assessing the impact of local contextual factors [1]. The British Medical Research Council (MRC) [1] has developed a research framework for complex interventions. They suggest a multi-step approach, including a development phase, followed by feasibility/piloting, evaluation (preferably through a randomised controlled trial) and implementation. They conclude that evaluation of complex interventions requires a mix of both quantitative and qualitative methods to get a more comprehensive understanding of the interventions. One challenge in complex interventions is the length and complexity of causal chains linking the intervention with outcome [1]. This means that the intervention may affect important factors that were not planned for and not measured with quantitative methods [1], factors that could be discovered with qualitative methods. Thus, knowledge from qualitative research of a complex intervention is crucial to fully interpret the results of and understand an intervention.

Case management is a complex intervention that has been used in different health care settings such as psychiatry and geriatric care [2,3]. It has no single definition but it has been suggested that basic case management may include identification and outreach, comprehensive individual-based assessment, care planning, care coordination, service provision, monitoring, evaluation and meeting individual needs [4,5]. Several studies have investigated the effects of case management for older people. These studies mainly focused on outcomes such as healthcare utilization and costs, quality of life, physical or cognitive functioning, quality of care and patient satisfaction [6]. The reported effects are contradictory [6]. In addition, such studies are seldom described in detail [7], which makes it difficult to compare them. Thus, there is a need for in-depth investigations to gain deeper understanding of the interventions, which will allow comparisons to be made and will enable us to draw conclusions about best practices [8]. To be able to further develop case management interventions a greater understanding of the intervention’s content and construction is needed. Qualitative studies are important to identify different barriers and facilitators that could be underlying reasons for an intervention being successful or not [9] and are necessary for implementation [10]. A qualitative evaluation is necessary to obtain a comprehensive description of the intervention’s components, to explore conditions for implementation, to establish construct validity and to facilitate possible replication [9]. Furthermore, it is essential to investigate different perspectives of the experience of an intervention, from both those receiving and those performing the intervention. However, studies of these different perspectives are generally lacking.

Some qualitative studies have focused on experiences of case management for older people [11-13]. The studies by JM Nelson and P Arnold-Powers [12] and K Brown, K Stainer, J Stewart, R Clacy and S Parker [11] found that the relationship between the case managers (CM) and participants was highly valued. JM Nelson and P Arnold-Powers [12] reported that the relationship with CM helped to provide security, safety and comfort for clients, and K Brown, K Stainer, J Stewart, R Clacy and S Parker [11] reported that their participants experienced that the CM had improved their quality of life. P Sargent, S Pickard, R Sheaff and R Boaden [13] and [11] also found that the participants were satisfied with the CMs’ different skills and ability to arrange services. According to P Sargent, S Pickard, R Sheaff and R Boaden [13] psychosocial support was emphasised by both the patients and carers with experience of case management, and was viewed as being equally important as clinical care. Complex interventions are dependent on the local context [1], which means that case management interventions could be experienced in various ways and have unique problems depending on the context they are performed in. Thus, each intervention needs to be explored in terms of what has been done and also how it was experienced, from both the providers’ and the receivers’ perspectives.

The aim was to explore older people's and case managers’ (CM) experiences of a complex case management intervention.

## Method

The study had a qualitative design, using opened-ended interviews with older people who were part of a case management intervention and the CMs who had performed the intervention.

### Participants

The study comprised 20 people: 14 participants (four men and 10 women, age 75-95 years, median age 83) who had received the case management intervention and six CMs (four nurses and two physiotherapists, age 31-51 years, median age 44) who had performed the intervention. Inclusion criteria for the older persons were: (1) age at least 65 years, (2) residence in an ordinary home, (3) need for help with two or more activities of daily living (self-reported and meaning that the participant could not perform the whole activity by them self, for example cleaning, transportation, and or managing medications), and (4) admission to hospital at least twice, or at least four visits to outpatient care, in the 12 months prior to entering the intervention study. In addition, participants had to be cognitively adequate and feel well enough to participate in an interview. Cognitive status was examined by using the Mini Mental State Examination (MMSE) [14]. The instrument covers cognitive areas of orientation, memory, attention, the ability to name, the ability to follow verbal and written commands, write a sentence spontaneously, and copy a complex polygon. Generally accepted cut-off points are; 25-30 for normal cognition; 21-24 for mild cognitive impairment; 14 or below for moderate or severe cognitive impairment [15]. In this study a cut-off of 25 points or higher out of a maximum of 30 required for participation. All participants included in the CM intervention had been recruited from a nearby university hospital, from the four primary care centres in the study municipality, through the municipal home care organisation or by the participants contacting the research group by themselves. The participants in the present study were recruited face-to-face by the research team during their participation in the CM intervention study. Purposeful selection [16] was used to obtain variation in gender and in age, use of home care services and CM (Table 1). Before entering the case management intervention study, the participants were informed, both in writing and verbally, that they might be asked about being interviewed with open-ended questions. This information was then repeated after nine months of the one-year intervention, when they were asked to participate in the interview.

**Table 1:** Characteristics of the interviewed receivers and providers of the intervention

**Receivers**				
**Code**	**Gender**	**Marital status**	**The interview was made after…**	**Participant receiving home care services**
R0005	Male	Widower	12 months	No
R0012	Female	Widow	13 months	No
R0020	Female	Widow	13 months	No
R0025	Female	Widow	13 months	No
R0026	Female	Divorced	14 months	Yes
R0029	Male	Living apart	14 months	Yes
R0031	Female	Widow	12 months	Yes
R0036	Male	Widower	12 months	No
R0053	Female	Married	15 months	No
R0079	Female	Widow	18 months	Yes
R0081	Female	Widow, living apart from a new man	16 months	Yes
R0083	Male	Widow	15 months	Yes
R0091	Female	Unmarried	13 months	No
R0143	Female	Married	12 months	No
**Providers**				
**Code**	**Profession of the CM**	**Code of the participant**	**Gender of the participant**	**Participant receiving home care services**
N0004	CM1 - Nurse	R0004	Female	No
N0025	CM2 - Nurse	R0025	Femalez	No
N0083	CM2 - Nurse	R0083	Male	Yes
N0055	CM3 - Nurse	R0055	Male	No
N0114	CM3 - Nurse	R0114	Male	No
N0086	CM4 - Nurse	R0086	Male	No
N0161	CM4 - Nurse	R0161	Female	No
P0028	CM5 - Physiotherapist	R0028	Female	Yes
P0081	CM5 - Physiotherapist	R0081	Female	Yes
P0085	CM5 - Physiotherapist	R0085	Male	No
P0095	CM5 - Physiotherapist	R0095	Female	Yes
P0098	CM6 - Physiotherapist	R0098	Male	No
P0134	CM6 - Physiotherapist	R0134	Female	No
P0151	CM6 - Physiotherapist	R0151	Male	Yes
P0169	CM6 - Physiotherapist	R0169	Female	No

Interviews were also conducted with the CMs. Six CMs were interviewed, two of whom had been educated as physiotherapists and four as nurses. The CMs were, depending on the number of participants, employed on a part-time basis in the research project. They were all recruited from municipal, primary care or hospital settings. The CMs were interviewed about every individual that they had met. In total, 162 interviews were made. Purposeful selection of fifteen interviews was used to obtain variation in CMs, gender, age and use of home care services. The CMs worked in the research project for between 2 and 5 years and had experience of caring for or rehablitating older people (Table 1).

### Setting

The case management intervention was in addition to standard care and the participants were consecutively recruited between 2006 and 2011. The intervention, conducted in the southern Sweden, was a one-year home-based case management intervention with home visits at least once a month [17]. The intervention comprised four components: *traditional case management* (including assessment, care planning, follow-up, care coordination, home visits, telephone calls and advocacy), *general information* (about the healthcare system, social activities, nutrition and exercise, among other things), *specific information* (related to the respondent’s specific health status, individual needs and medication) and *safety and continuity* (availability of CM by cell phone during working hours) [17].

The CM study was developed according to the MRC’s framework for complex interventions [18]. The pilot study phase, in which the intervention was developed, is described elsewhere [17]. Changes after the pilot study have also been reported [19].

### Data collection

Data were collected by means of personal interviews. The interviews were conducted between 2007 and 2012, and were conducted by four different persons due to a change in staff during this period. The first author (M.S.), the fourth author (J.K.), and two research assistants (one male and one female) conducted eleven, eight, eight and two interviews, respectively. The interviews were semi-structured, which meant that they were neither fully structured nor fully unstructured. The participants were free to talk about any subject, but the interviewer guided the interview [20]. Two thematic interview guides were used – one for the participants and one for the CMs – to ensure that the interviews covered the same areas of content. The CM interviews covered two themes: (1) the person they met and how the contact started, what they had done and what effects they thought this might have had; and (2) how they perceived the intervention, whether there was something that they considered successful or unsuccessful. The interview guide for the participants did not only comprise questions about the intervention: as well as questions on “help and support” (including questions about the CM and the case management intervention), it also covered “health”, “contacts with the healthcare system” and “the future and concerns”. Open questions were used and included questions such as “could you tell me about an ordinary meeting with the case manager?” (to the participant) and “could you tell me about this person that you have met in your role as case manager?” (to the case manager). Probing questions could for instance be “could you give an example?”, “how did that feel?” and “What did you do then?”. The interview guides were changed slightly during the study meaning that the order of the questions where changed, and thus all interviews covered the same areas. All interview guides were tested in pilot interviews on both the participants and CMs. No major changes were made in the interview guides after the pilot interviews and thus included in the study. Each interview started with clarification of the aim of the interview and the interviewee’s right to terminate the interview whenever he/she wanted.

The interviews with participants were conducted after they had received the intervention for at least nine months in order that they had undergone the majority of the intervention. They were interviewed after a mean of 14 months after they were included in the CM intervention. The interviews were carried out in a place chosen by the participant. All interviews took place in the participants’ homes and were between 40 minutes and 2 hours 51 minutes long. During the interviews, no-one besides the participant and the interviewer was present. However, in one interview the sister was in an adjacent room and the participant asked her some questions.

Interviews with the CM were made for each participant they had met after the participant had received the intervention for at least nine months. The CM interviews were conducted after in mean 17 months after the participant had been included in the intervention study. The CMs had with them the case records of their participants. All CM interviews took place at the department of the researchers and lasted between 9 and 24 minutes. All interviews were audio recorded and transcribed verbatim.

### Analysis

The interviews were analysed by content analysis. The analysis was influenced by B Berg [21], who suggests that content analysis may comprise a combination of both manifest and latent analysis. The manifest part concerns what is said and is visible in the text, while the latent part concerns finding an interpretable structure, a deeper underlying meaning [21]. The analysis was made using different steps inspired by UH Graneheim and B Lundman [22]. In the first step, the transcribed interviews were read several times independently by all authors to obtain a sense of the whole. In the second step, meaning units related to the aim were identified from the text. The third step involved condensing the meaning units into codes. The next step embraced a movement between the meaning unit and the text, between the text as a whole and its parts. During this process, subcategories and categories were identified. Three interviews were analysed independently by the first (M.S.) and last (J.K.) authors. The subcategories were then discussed by M.S. and J.K. until a consensus was reached. The first author then analysed an additional number of interviews and the subcategories were again discussed by M.S. and J.K. Groups of subcategories sharing the same content were arranged under tentative categories. The remaining interviews were divided between M.S. and J.K. and analysed independently. M.S. and J.K. discussed the content of the subcategories and developed categories. Finally, the four authors discussed the findings until a consensus was reached, and additional small adjustments were made to the categories. Quotations were chosen to illustrate the different subcategories. Examples of the analysis process are presented in Table 2.

**Table 2:** Examples of the analysis process

**Interview**	**Meaning unit**	**Code**	**Subcategory**	**Category**
N0086	He had not really the insight that there was something seriously… but he just laughed it off when you talked about it.	A failure to reach	Dealing with barriers	ENTERING A NEW PROFESSIONAL ROLE
P0028	We talked about it… about residential care for her. And if it… tried… well, talk a bit about what it was like to have some people around and so. But she… no. She did not want to. She did not want much at all [laughs a bit].	Meeting people that do not want to be helped or do not want to incommode		
N0025	You become despondent when you do not succeed. But … but I have offered it anyway. It sure is tough, so, it is.	To feel personal involvement	Setting limits	
R0031	Yes we're talking about everything … I think. I do not remember anything exactly… but we have… we are talking about everything. Yes it is just as if we have become friends. I see it as if she has become my friend (pause).	To feel confident in a person and her competence	Reliable competence	A POSSIBLE ADDITIONAL0020RESOURCE
R0079	And it's never in a hurry either, but they… There was never any hurry. Never ever. And they were helpful.	To get a chance to build a stable relationship		
R0143	Well you, that (pause) that I have not needed to search for [health care] because all I have needed… uh to ask for uh I have uh used the case manager for that…	To find a replacer for the usual health system	Gaining a safety net	

### Ethical considerations

This study was approved by the Regional Ethical Review Board in Lund (Ref. nos. 342/2006 and 499/2008) and is registered at Clinicaltrails.gov (Ref. No NCT01829594). All participants provided written informed consent for participating. The participants’ autonomy was acknowledged by emphasising, both before and at the beginning of the interview, that participation was voluntarily, and that the participant could withdraw from the study at any stage. They also were also informed that confidentiality should be maintained when presenting the results.

## Results

The experience of the intervention was interpreted from two perspectives: that of the CMs (i.e. the providers) that of the older persons (i.e. the receivers). This gave two content areas: (1) providing/receiving case management as a model and (2) working as, or interacting with, a CM as a professional. The results constituted of four categories: (1 and 2) the case manager as a coaching guard and case management as entering a new professional role, as seen from the provider’s perspective; and (3 and 4) the case manager as a helping hand and case management as a possible additional resource, as seen from the receiver’s perspective. The categories comprise various subcategories reflecting their content (Table 3).

**Table 3:** Main categories and categories

	**The provider’s perspective**	**The participant’s perspective**
***The case manager as…***	***A coaching guard***	***A helping hand***
	**• The solver**	**• The one who has information**
	**• The supporter**	**• The one who supports**
	**• The standing guard**	**• The one who keeps an eye on things**
	**• The navigator**	**• The one who knows what to do or where to turn**
***Case management as…***	***Entering a new professional role***	***A possible additional resource***
	**• Dealing with barriers**	**• Something unknown**
	**• Building trust**	**• Reliable competence**
	**• Setting limits**	**• Limited resource**
	**• Making a possible difference**	**• Gaining a safety net**

### The provider’s perspective

#### The case manager as a coaching guard

One category concerned the providers’ view of themselves as coaching guards in their roles as CMs. This implied functioning as someone who solved problems, someone who supported the participant and helped them when something happened, as well as helping the participant to navigate through the health system in terms of contacting and interacting with various caregivers. It also implied being a guard that could take control if the situation required it. The category covered four different subcategories: “The solver”, “The supporter”, “The standing guard” and “The navigator”.

##### The solver

The CMs solved various kinds of problems. The text revealed that this could both be problems linked to the participant’s health status but also practical matters such as helping participants to put up shelves. It was also shown that the CMs used different strategies to identify problems, either by conducting structured assessments or examinations, or simply by asking. They were also attentive to changes in the participant’s situation and asked questions in areas where they thought there could be problems. Problems detected included problem with balance, pain and loneliness. The CMs tried to solve the problems using various strategies, for example, encouraging a participant they identified as being lonely to take part in social events or arranging contact with a voluntary organisation. Problems could also be solved by giving information, answering questions or introducing a pain diary so that the participant would get better pain control. For some participants, the CM developed individualised training programs focusing on, for instance, balance, physical activity or breathing. It was also shown that the CM followed up their actions to determine their consequences. This could be reassessment or a statement from the participant that they had less pain.

*Last time we were there he again had much swelling in … yes, his feet, and we said that he should probably contact a doctor immediately because of this. And they had actually done this and had been at the primary care center with … a doctor, and the neighbor had* … *driven him because his daughter was on holiday. And he had had some changes to his diuretic medication and*, *um* (*pause*), *his feet looked much better the last time we were there* … (*N0114*)

##### The supporter

When participants did not manage to solve things entirely by themselves, the CM needed to give support. Support could be trying to motivate the participant or their next of kin to take part in the actions the CM had initiated. The CMs experienced that they sometimes had pushed the participant in the right direction, had convinced or coached the participant. They could also give support to next of kin so they would be able to encourage the participant to do, for instance, their exercises. Support was expressed in terms of practical, social or emotional support. Practical support could, for example, be accompanying the participant to the hospital and social support was shown when the CM was someone the participant could talk to and to discuss everyday questions with. Emotional support could be to talk to the next of kin and ease their burden of living with a frail older person. It could also be to live talk with and comfort the participant.

*She* … *had a very strong need to talk about her situation* … *so the first few times it was enough to just sit and talk with her*. (*P0095*)

##### The standing guard

When the CMs described themselves as a standing guard it meant offering defence or security and being someone who monitored and had an overview of the participant’s situation. One aspect was that it was seen to be important to be responsive to the participant’s interests and to be able to be a spokesperson and advocate if needed. To be a standing guard also included that the CMs expressed that the participants or their next of kin, contacting them at unplanned times with urgent issues. The CMs also experienced that they themselves were a source of security and that they had an active role in keeping track and could react if anything happened.

*And so I tried to emphasise to the* [*physician*] *that it was not just that she was dizzy*, *but that she actually fell. It would sooner or later end badly*. (*P0081*)

##### The navigator

When the CMs were in contact with other agents in the healthcare system they acted as navigators. The CMs expressed that they informed the participants about the care system and helped them with bureaucracy and other barriers. This could, for example, be helping the participant with an application form for transportation services or debt settlement. Navigation could also involve formal or informal caregivers and be direct contact initiated by the CM, but it could also be advising the participants where to go or whom to contact when they needed help. The latter was common since the participant often did not know what help they could get or whom to contact.

*R*: (…) *his wife had to help him up five or six times a night and then there was nothing* – *yes there was a crisis there for a while* …

*I*: *And what did you do about it*?

*R*: *I contacted the occupational therapist and physiotherapist so that he got help with it*, *and so that he got this period of relief* (*P0098*)

#### Case management as entering a new professional role

This category covered four subcategories: “Dealing with barriers”, “Building trust”, “Setting limits” and “Making a possible difference”. It was obvious that when the CM narrated about their experiences of case management it was in the light of working in new ways. The new role entailed both possibilities and barriers. The barriers included sometimes failing to reach the participant and therefore failing to help them or to perform the intervention. The possibilities concerned building stable and good relationships with the participants and the intervention enabling them to make a difference in terms of helping people. To work with a model that sometimes included strong relationships could also be challenging as the CMs sometimes felt personally involved and therefore had to define and set limits for the relationships.

##### Dealing with barriers

Sometimes the CMs could not perform the intervention as they wanted. One barrier was that they sometimes reported that they were powerless and that they were not acknowledged by the healthcare agencies, that they did not get any help or response from them. Some participants did not have any expectations or tasks that they wanted the CM to take care of, which was also experienced as a barrier. Another barrier was that the CMs experienced that the participants did not have any major problems that they could solve. The CMs also experienced that some participant did not follow the advices they were given because the participants had high expectations that were hard to fulfil, did not want to be a burden or did not want be helped.

*He* … *did not want to let me in*, *but instead said*: “*Yes*, *you can come when you have arranged these things*.” *But they were things I couldn*’*t help to arrange*. (*N0055*)

##### Building trust

The CMs expressed that they had to build trust with the participant to be able to perform and implement the intervention. The CMs described trying to be frank and truthful when talking with the participants in order or to build trust and felt that it was crucial to treat them with respect. One CM said:

(…) *and it is clear that she could have got more help but* … *but she really wanted to struggle on herself. This business of dressing as well* … *It would certainly have been much easier if you dressed her*, *instead of sitting for twenty to thirty minutes every morning. But she really wanted to do it*. (*P0095*)

When the participant was motivated and, for instance, fought and struggled with exercises, the CM felt that they were trusted and that this trust was built upon a mutually respectful relationship. The CM also experienced that different factors were important to gain this trust, such as time, continuity and personal chemistry. The confidence – often expressed as mutual confidence – was something that the CM valued highly and was seen as an important part of the intervention.

*Yes*, *after the first meeting*, *after the first few minutes in fact*, *it felt so right. Partly because R* [*the participant*] *is a very open person who can talk about their life quite openly. Yes*, *our first contact was actually very good*, *and it still feels really good*. … *Our contact has been very positive* (*N0004*)

##### Setting limits

Working in a new professional role also implied that the CM had to work in new ways, interact with participants and tackle other problems than they had faced in their professions. This sometimes resulted in strong personal involvement that had to be dealt with. When the CMs got personally involved they felt that it could be difficult saying no to the participants. It was shown that they sometimes did things that could be considered going beyond their duties, for instance, calling the participant late at night. The need to set limits was also shown when the CMs expressed personal involvement and feelings about the participants and the intervention. The need to set limits was sometimes experienced as a difficulty in ending the intervention. The CMs experienced feelings of guilt and stated that they were worried about what would happen with the participant when their visits ceased. The CMs did not always know how to set limits. This could, for example, be when the next of kin interrupted the conversation or answered instead of the participant. The CMs expressed wishes and thoughts about the intervention and the skills they would have needed to deal with certain situations, for example, expertise in motivational interviewing. The text also revealed frustration when the participant did not do what was agreed, guilt and anguish when they had given some advice that did not turn out to be right and anxiety if the participant for some reason became upset. To deal with this, the CM had to set limits to reduce their involvement and the personal responsibilities that they felt. The CMs sometimes justified not performing a certain action by pointing out the irrationality of doing it.

*Well*, *then I felt a bit guilty because I could easily have done it* [*gone to the bank with the participant*’*s money when she was hospitalised*]. *But you can*’*t get too involved as it becomes hard to say no. And she puts all her trust in me*. (*N0004*)

##### Making a possible difference

One important aspect of the case management function was that it was experienced as being useful and something that could make a possible positive difference. This could be seen when the CMs met participants who been ignored by the ordinary health system or who had not been listened to or helped in spite of their problem having been pointed out several times by themselves or their relatives. This could also be related to the different parts the intervention was planned to include, such as information, education and different exercises.

*I*: *But did you feel that she benefited from what you did for her*?

*R*: *Yes*, *the pain diary* [*shows*], *above all*, *that she had less pain. That was great. And she took her medications more regularly* (*P0169*)

### The receiver’s perspective

#### The case manager as a helping hand

From the participant’s perspective the contact with the CM was very much about receiving a helping hand. A helping hand that could hold them tight when they needed it, lead them through difficulties and pointing out the direction when they were confused. A helping hand could also be supportive when they felt they were losing control. The category covered four subcategories: “The one who has information”, “The one who supports”, “The one who keeps an eye on things” and “The one who knows what to do or where to turn”. These categories are closely linked to each other as, for example, someone who knows where to turn is also someone who has information.

##### The one who has information

The participants expressed that when they had questions they could turn to the CM as a source of information. They felt that the CM had the right information and could solve their problem. When participants needed information they also needed to get in contact with the CM, and they expressed feeling able to contact the CM to get answers to their questions.

*I*: (…) *have they* [*the case managers*] *made a difference for you*, *do you think*?

*R*: *Yes*, *a little bit* … *anyway. Because*… *you can ask about things. If there is anything you need to know*, *they can give you the answer*. (*R0083*)

##### The one who supports

In the same way as the CM experienced that they could give different kinds of support, the participant also experienced being supported in various ways. They experienced practical support from the CM when they, for instance, needed to go to the pharmacy or needed help buying new spectacles. The text also revealed that the CM was an important source of social support. They experienced that the CM interacted socially with them and that it was good to have someone to talk to, and expressed enjoying having a CM. The text also revealed that support was also about the attitude of the CM, i.e. the CM being positive and easy-going. Another characteristic that was experienced to be important was the CM’s ability to motivate and be a prod.

*Yes*, *they tried to persuade me to be X*-*rayed and to think about surgery. Tried to persuade me to* … *do the things that I tend to put off. I always put things off*, *there is so much to choose from so I* … *put it off and it doesn*’*t get done*. (*R0091*)

##### The one who keeps an eye on things

When the CM followed up and checked things, asked and made sure that everything was proceeding as planned, the participant felt that someone was keeping an eye on things. This included the CM asking questions and performing examinations, e.g. measuring blood pressure and testing balance. The participants were aware that the CMs made notes and filled in forms about their health status and that this was a form of surveillance. It was important to meet the CM regularly and when they felt that they needed them in order to experience that they were keeping an eye on things. The participants expressed that the CM visited them at least once a month, at home or at the hospital, and made telephone calls, but felt that they still had some influence over the amount of contact. Another aspect of keeping an eye on things was that they felt that the CM was someone who performed examinations in order to be able to intervene if necessary.

*Because she looked at that* [*leg*] *every time she came*, *because you know it*’*s still not quite right*, *because it*’*s still swollen and a bit red. But she said that it*’*s* … *the warfarin*. (*R0025*)

##### The one who knows what to do or where to turn

The participants experienced that the CM knew what to do or where to turn when they used the CM’s knowledge on a specific occasion. It was expressed that participants got advice from the CM as to where to turn in the healthcare system when they felt ill or if they had a problem. The participants described the CM giving advice about what to do about a specific problem or a specific training program. Some participants expressed that the CMs helped them to fix a wide range of things, ranging from specific problems to things that the participants had not experienced as problems.

*And I find it very difficult to keep my balance. And they* [*name*, *physiotherapist in the project*] *asked me how would it be if you stood with your legs further apart* … *then your balance will be a bit better* … *And I*’*ve been doing it*, *and it*’*s absolutely true*, *because now I can stand and wash up* … (*R0079*)

#### Case management as a possible additional resource

For the participants, case management was something new. Initially, they did not know exactly what to expect or what they could use the resource for. In most cases, the participants eventually realised that case management included a competence that one could depend on. In some cases, a strong relationship was built and case management was experienced as something beneficial and something that could contribute to a sense of security. The participants knew that the case management was a limited resource, in terms of both in intensity and time. But they experienced case management as a resource that sometimes replaced usual care, and a resource not only for practical matters, but also emotional ones. The category comprised four subcategories: “Something unknown” “Reliable competence”, “Limited resource” and “Gaining a safety net”.

##### Something unknown

To enter “Something unknown” could mean that the participants did not always understand the purpose of the intervention or why the CMs wanted them to do certain things. This was expressed by some participants as they did not know what the CM could do, did not want to be helped, or wanted to manage their problems on their own.

*I*: *Has this meant anything to you*? *Has it been important* [*R interrupts*]

*R*: *I didn*’*t really know what it meant*, *but it*’*s clear that uh* … *she was like* (*mumbles a little*) *some support anyway*, *after all* (*pause*) (*R0029*)

##### Reliable competence

When the participants got the help and information they needed they felt confident in the CM intervention. This confidence could contribute to a strong relationship between the CM and the participant. The text revealed different aspects of what the participant experienced as important to build a fruitful relationship. It was experienced that interpersonal aspects such as personal chemistry and a good connection were important, but also aspects of the intervention design, such as length of the visits and continuity. Some also expressed that they were a bit hesitant before they knew what the intervention was all about, but that this changed when they got to know the CMs. Case management as a reliable competence was showed when the participants felt that they had become friends with the CM and when they expressed that they knew that the CM could solve specific tasks. The text revealed that the participants could talk to the CM about things that they did not want to discuss with someone else.

*And you could talk to her* … *about everything. About things I do not want to mention to you. But I developed very good trust in her*… (*R0143*)

##### Limited resource

When a strong and stable relationship with the CM ceased after one year, some of the participants felt a sense of loss. Even though they knew that the project would end someday, they experienced it as a limited resource. The participants expressed strong feelings for the CMs and their whereabouts and they expressed that it was hard to end their participation in the project. Many participants expressed that they missed the CMs and their visits.

*R*: (…) *I miss her when she doesn*’*t come*.

*I*: *You*’*ve missed it*?

*R*: *Yes*, *it was a bit tough* … *a very sweet person I must say*… (*R0012*)

##### Gaining a safety net

To be a part of an intervention that provided contact and visits on a regular basis made the participants feel secure and this contributed to them gaining a safety net. The feeling of a safety net was also built by the participants’ ability to be helped by the intervention. Many participants had benefited from the advice or the actions of the CM. This was expressed both in specific areas such as pain or walking ability, but also in a more general way when they expressed that the intervention as being very good or perfect. They also expressed awareness that the intervention could help them if they had a question or if they were to become ill, despite them having no problems at this time.

*I feel secure* … *just knowing she will come*, *now I have the time she will come*, *now I think it might be the nineteenth of* … *May she will come*, *no the nineteenth of June*, *I looked at the calendar the other day and you have put a mark in it. Just knowing she will come makes me feel secure*. (*R0031*)

## Discussion

### Discussion of the results

There is a need to investigate the ‘black box’ of complex interventions and this study was important to be able to understand some of the essential components in this case management invention. The knowledge we have gained could contribute to understanding of the possibilities and challenges in complex case management interventions and implementation.

It was obvious that trust and confidence were crucial facilitators for performing the intervention and they permeated various categories. It is known that trust and confidence are important factors for building and maintaining a solid relationship between patients and caregivers [23-25] and are particularly important for older people with repeated health care contacts. A good relationship has been reported to improve health outcomes [26], as well as perceived effectiveness of care and self-reported health [27]. In addition, previous case management studies showed that the CM-client relationship is important for enabling the CM to provide help [12]. Interpersonal continuity and patient/person-centred care have been suggested to be important factors for establishing a strong relationship [23,28]. This is, however, not always achieved in the regular health system [29-31]. It is possible that the person-centred approach and regular visits/repeated contacts were two facilitators that contributed to the solid CM-client relationship in this intervention. The findings suggest that the case management intervention may compensate for shortcomings in the existing system in terms of being more person-centred and thus encouraging a strong trusting relationship. Client-centeredness is also one component that has been stated to be one of the theoretical components of case management [5] and positive effects on client/patient-centeredness have also been found in a previous review investigating case management in primary care [32].

Another important finding was the CMs’ ability to meet participants’ unmet service needs. “The solver”, “The standing guard” and “The one who keeps an eye on things” were important categories contributing to this result. The improvement in identifying unmet service needs through case management was reported in a review by EC You, D Dunt, C Doyle and A Hsueh [33] and in a Canadian study by R Hebert, M Raiche, MF Dubois, NR Gueye, N Dubuc and M Tousignant [34]. The ability to meet unmet needs and to react if anything happens also contributed to feelings of security among the participants in this study. This is in line with other studies that reported that both clients and CMs felt that the ability to detect health-related changes in the receivers’ conditions contribute to a feeling of security [11,13,35]. The feeling of security reported in this study stands in contrast to insecurity that dependent older people may experience in the ordinary health system due to reduced autonomy, limited possibilities for negotiation [36] or fear that they will get abandoned by the carer and not receive any care if they criticise the care [37,38]. Thus, the CMs’ ability to help and solve problems and give support when needed seems to be a fundamental CM function in this study. This function was also important for the participants as it contributed to a feeling of security, which is highly desirable in healthcare as it a prerequisite for successful care.

The challenges the CMs met in terms of undergoing a transition from a familiar profession to a CM role was another important finding in the present study. This was seen when the CMs felt too personally involved and had to set limits. This may be a result of the flexibility within their role as CMs which allowed them to be creative and find individual solutions for the participants. This flexibility and free role could also be problematic. The lack of role definition has been identified as a key barrier to the success of case management for older people [39]. Thus, flexibility may be a facilitator for finding individual solutions for the clients, but may at the same time be a barrier in terms of making the role unclear. It was obvious that the CM underwent a transition to deal with this new way of working. This process could be difficult and could make the CMs vulnerable and subject to various kinds of stressors [40]. It is therefore important to give the CMs solid support to deal with the professional transition and thus the difficulties setting limits [41]. One form of support could be mentorship/supervision [42]. When implementing case management the CMs should also receive training in a multidisciplinary collaboration [35,41]. This could increase awareness of the CMs by other practitioners in the health system, which is important to be able to improve the outcomes of the role [35]. Thus, when implementing a case management intervention, efforts should be made to support the CM and to acknowledge the case management function in the existing health system.

### Study limitations

Qualitative studies can be assessed through the concept of trustworthiness, which comprises credibility, transferability, dependability and conformability [43]. Credibility refers to the believability of the data and whether the findings are faithfully based on the descriptions provided by the participants [43]. To deal with potential threats to credibility, data from both older persons and CMs were used. To increase credibility, efforts to achieve variation were made (Table 1). One potential threat to credibility was the length and, thus, depth of the interviews. However, the CMs knew the aim of the interview and what they were supposed to talk about, which made the interviews very focused. In the interviews with the participants, only a small part of the interview was about the experiences of the intervention, which may also have influenced the depth. In qualitative research, the sample size should be based on the information needs [16]. The concept of data saturation – sampling until no new information is obtained and redundancy is achieved – is widely used. No new subcategories emerged when analysing the final ten interviews, which allowed us to conclude that saturation was reached DF Polit and CT Beck [16]. Dependability refers to whether the interpretations are representative and whether the data are stable over time [43]. To strengthen dependability, the interviews were carried out by different persons and thematic interview guides were used to make sure the interviews covered the same areas. Having different interviewers also strengthens the conformability, which relates to questions about the researches’ subjectivity. To reduce the risk of the results being influenced by the researches’ preunderstanding, investigator triangulation [16] was used. Concerning the transferability of the results, it is important to bear in mind that the local context may have influenced the findings.

## Conclusion

The experiences of a case management intervention could be interpreted from the CM perspective as entering a new professional role and being a coaching guard for the older person. The older persons experienced the intervention as receiving a possible additional resource and the interaction with the CM as a helping hand. The new professional role could be experienced as both challenging and as a barrier. Continuous professional support is seemingly needed when implementing a case management intervention for older persons. Mutual confidence and the participants experiencing trust, continuity and security were important elements and an important prerequisite for the case manager to perform the intervention and make a difference. It was obvious that the some older persons had unfulfilled needs that the ordinary health system was unable to meet. The CM was seemingly able to fulfil some of these needs and was experienced as a valuable complement to the existing health system.

## Competing interests

The authors declared that they have no competing interests.

## Authors’ contributions

MS participated in the design of the study, in the collection of data, performed the analysis and interpretation of data, and drafted the manuscript. PM participated in the design of the study and helped in the analysis of the data and to draft the manuscript. UJ participated in the design of the study, participated in the design of the study and helped in the analysis of the data and to draft the manuscript. JK participated in the design of the study, participated in the data collection, performed the analysis and interpretation of data, and helped in the drafting of the manuscript. All authors read and approved the final manuscript.

## Pre-publication history

The pre-publication history for this paper can be accessed here:

http://www.biomedcentral.com/1472-6963/14/14/prepub

## References

[B1] Developing and evaluating complex interventions: new guidance http://www.mrc.ac.uk/complexinterventionsguidance

[B2] Kurtz LF Bagarozzi DA Pollane LP Case management in mental health Health Soc Work 1984 14 3 201 211 6479752 10.1093/hsw/9.3.201

[B3] Toseland RW O'Donnell JC Engelhardt JB Hendler SA Richie JT Jue D Outpatient geriatric evaluation and management. Results of a randomized trial Med Care 1996 14 6 624 640 10.1097/00005650-199606000-00011 8656727

[B4] Björkman T Case management for individuals with severe mental illness: a process-outcome study of ten pilot services in Sweden PhD thesis 2000 Lund University: Department of Clinical Neuroscience

[B5] Case Management Society of America Standards of Practice for Case Management 2010 3 Little Rock: Case Management Society of America

[B6] Low LF Yap M Brodaty H A systematic review of different models of home and community care services for older persons BMC Health Serv Res 2011 14 93 10.1186/1472-6963-11-93 21549010 PMC3112399

[B7] Eklund K Wilhelmson K Outcomes of coordinated and integrated interventions targeting frail elderly people: a systematic review of randomised controlled trials Health Soc Care Community 2009 14 5 447 458 10.1111/j.1365-2524.2009.00844.x 19245421

[B8] Markle-Reid M Browne G Weir R Gafni A Roberts J Henderson SR The effectiveness and efficiency of home-based nursing health promotion for older people: a review of the literature Med Care Res Rev 2006 14 5 531 569 10.1177/1077558706290941 16954307

[B9] Farquhar MC Ewing G Booth S Using mixed methods to develop and evaluate complex interventions in palliative care research Palliat Med 2011 14 8 748 757 10.1177/0269216311417919 21807749

[B10] Grol R Baker R Moss F Quality Improvment Research: Understandning the science of change in health care 2004 London: BMJ Books 10.1136/qhc.11.2.110 PMC1743590 12448794

[B11] Brown K Stainer K Stewart J Clacy R Parker S Older people with complex long-term health conditions. Their views on the community matron service: a qualitative study Quality in primary care 2008 14 6 409 417 19094416

[B12] Nelson JM Arnold-Powers P Community case management for frail, elderly clients: the nurse case manager's role J Nurs Adm 2001 14 9 444 450 10.1097/00005110-200109000-00011 11561425

[B13] Sargent P Pickard S Sheaff R Boaden R Patient and carer perceptions of case management for long-term conditions Health Soc Care Community 2007 14 6 511 519 10.1111/j.1365-2524.2007.00708.x 17956403

[B14] Folstein MF Folstein SE McHugh PR "Mini-mental state". A practical method for grading the cognitive state of patients for the clinician J Psychiatr Res 1975 14 3 189 198 10.1016/0022-3956(75)90026-6 1202204

[B15] Mungas D In-office mental status testing: a practical guide Geriatrics 1991 14 7 54 58 63, 66 2060803

[B16] Polit DF Beck CT Nursing research: generating and assessing evidence for nursing practice 2012 9 Philadelphia: Wolters Kluwer Health/Lippincott Williams & Wilkins

[B17] Kristensson J Ekwall AK Jakobsson U Midlov P Hallberg IR Case managers for frail older people: a randomised controlled pilot study Scand J Caring Sci 2010 14 4 755 763 10.1111/j.1471-6712.2010.00773.x 20409057

[B18] Medical Research Council A framework for development and evaluation of RCTs for complex interventions to improve health 2000 London: Medical Research Council

[B19] Sandberg M Case management for frail older people. Effects on healthcare utilisation, cost in relation to utility, and experiences of the intervention PhD thesis 2013 Lund University: Department of Health Sciences

[B20] Kvale S Brinkmann S Interviews, Learning the craft of qualitative research interviewing 2009 2 London: SAGE Publications

[B21] Berg B Qualitative research methods for the social sciences 2004 Boston: Allyn and Bacon

[B22] Graneheim UH Lundman B Qualitative content analysis in nursing research: concepts, procedures and measures to achieve trustworthiness Nurse Educ Today 2004 14 2 105 112 10.1016/j.nedt.2003.10.001 14769454

[B23] McCormack B Person-centredness in gerontological nursing: an overview of the literature J Clin Nurs 2004 14 3a 31 38 15028037 10.1111/j.1365-2702.2004.00924.x

[B24] Mechanic D The functions and limitations of trust in the provision of medical care J Health Polit Policy Law 1998 14 4 661 686 9718518 10.1215/03616878-23-4-661

[B25] Thorne SE Robinson CA Reciprocal trust in health care relationships J Adv Nurs 1988 14 6 782 789 10.1111/j.1365-2648.1988.tb00570.x 3230220

[B26] Lee YY Lin JL Linking patients' trust in physicians to health outcomes Brit J Hospital Med (London, England : 2005) 2008 14 1 42 46 10.12968/hmed.2008.69.1.28040 18293731

[B27] Hall MA Dugan E Zheng B Mishra AK Trust in physicians and medical institutions: what is it, can it be measured, and does it matter? Milbank Q 2001 14 4 613 639 v 10.1111/1468-0009.00223 11789119 PMC2751209

[B28] Waibel S Henao D Aller MB Vargas I Vazquez ML What do we know about patients' perceptions of continuity of care? A meta-synthesis of qualitative studies Int J Qual Health Care 2012 14 1 39 48 10.1093/intqhc/mzr068 22146566

[B29] Baker R Boulton M Windridge K Tarrant C Bankart J Freeman GK Interpersonal continuity of care: a cross-sectional survey of primary care patients' preferences and their experiences Br J Gen Pract 2007 14 537 283 289 17394731 PMC2043338

[B30] McCormack B Karlsson B Dewing J Lerdal A Exploring person-centredness: a qualitative meta-synthesis of four studies Scand J Caring Sci 2010 14 3 620 634 10.1111/j.1471-6712.2010.00814.x 21050249

[B31] Preston C Cheater F Baker R Hearnshaw H Left in limbo: patients' views on care across the primary/secondary interface Qual Health Care 1999 14 1 16 21 10.1136/qshc.8.1.16 10557664 PMC2483626

[B32] Ferguson JA Weinberger M Case management programs in primary care J Gen Intern Med 1998 14 2 123 126 10.1046/j.1525-1497.1998.00029.x 9502373 PMC1496915

[B33] You EC Dunt D Doyle C Hsueh A Effects of case management in community aged care on client and carer outcomes: a systematic review of randomized trials and comparative observational studies BMC Health Serv Res 2012 14 395 10.1186/1472-6963-12-395 23151143 PMC3508812

[B34] Hebert R Raiche M Dubois MF Gueye NR Dubuc N Tousignant M Impact of PRISMA, a coordination-type integrated service delivery system for frail older people in Quebec (Canada): A quasi-experimental study J Gerontol B Psychol Sci Soc Sci 2010 14 1 107 118 10.1093/geronb/gbp027 19414866

[B35] Russell M Roe B Beech R Russell W Service developments for managing people with long-term conditions using case management approaches, an example from the UK Int J Integr Care 2009 14 e02 19340326 10.5334/ijic.303 PMC2663703

[B36] Ellefsen B Dependency as disadvantage - patients' experiences Scand J Caring Sci 2002 14 2 157 164 10.1046/j.1471-6712.2002.00073.x 12000669

[B37] Strandberg G Astrom G Norberg A Struggling to be/show oneself valuable and worthy to get care. One aspect of the meaning of being dependent on care–a study of one patient, his wife and two of his professional nurses Scand J Caring Sci 2002 14 1 43 51 10.1046/j.1471-6712.2002.00053.x 11985748

[B38] Strandberg G Norberg A Jansson L Meaning of dependency on care as narrated by 10 patients Res Theory Nurs Prac 2003 14 1 65 84 10.1891/rtnp.17.1.65.53170 12751886

[B39] Chapman L Smith A Williams V Oliver D Community matrons: primary care professionals' views and experiences J Adv Nurs 2009 14 8 1617 1625 10.1111/j.1365-2648.2009.05002.x 19456996

[B40] Schumacher KL Meleis AI Transitions: a central concept in nursing Image J Nurs Sch 1994 14 2 119 127 10.1111/j.1547-5069.1994.tb00929.x 8063317

[B41] Reimanis CL Cohen EL Redman R Nurse case manager role attributes: fifteen years of evidence-based literature Lippincotts Case Manag 2001 14 6 230 239 quiz 240-232 10.1097/00129234-200111000-00002 16398069

[B42] Rosser M King L Transition experiences of qualified nurses moving into hospice nursing J Adv Nurs 2003 14 2 206 215 10.1046/j.1365-2648.2003.02695.x 12834379

[B43] Lincoln YS Guba EG Naturalistic Inquiry 1985 Newbury Park, CA: Sage Publications

